# A Study on Students Acquisition of IT Knowledge and Its Implication on M-Learning

**DOI:** 10.1155/2015/248760

**Published:** 2015-10-21

**Authors:** A. Balavivekanandhan, S. Arulchelvan

**Affiliations:** Anna University, Chennai, India

## Abstract

The boom in mobile technology has seen a dramatic rise in its usage. This has led to usage of mobiles even in the academic context for further learning. Although the advantages of m-learning (mobile learning) are visible, studies are required to address the aspects that shape its virtual expectations. The acceptance of mobile technology relies mostly on how the students feel about mobile technology fitting into their requirements. Yet, in spite of the significance in the potential of m-learning, research studies have only inadequate data to identify the factors that influence their decision to adapt the mobile technology for the purpose of learning. To deal with this space, the present study was undertaken to correlate the IT skills of students with their impact on their acceptance of m-learning. The research study found that the perceived usability along with the usefulness of m-learning impacts the association between IT expertise and the objective of learners' acceptance of m-learning. A survey of 892 students from Engineering, Arts, and Science Colleges found that IT skills influence student's acquisition of m-learning technology. Specialized and advanced skills in mobile technology along with basic skills play a significant role in influencing a student to accept m-learning. But no specific substantiation has been established to support the statement that highly developed IT skills have influenced the students to accept m-learning.

## 1. Introduction

Mobile technology has expanded its perimeter into the educational sphere as a means to facilitate learning that is not limited by time and place. Existing research has focused on the ways in which mobile technology is utilized by scholars and teachers [1–3]. There is a need, therefore, for research that focuses on the factors which influence the acceptance and implementation of m-learning. The acceptance of mobile technology by learners and instructors happens only when they trust that m-learning suits their exact requirements. The choice to implement m-learning is influenced by many factors. Of these factors, the one that has received inadequate notice is the impact that learners' information technology (IT) proficiency and understanding have on their acceptance of mobile as a learning medium. In those researches that have studied this association, IT expertise was employed to a larger extent that does not permit of likely contradistinctive effects from the diverse forms of IT skills [4–6].

Numerous studies have made efforts to evolve a theoretical context or a prototype to explore and evaluate m-learning [7]. The theoretical context helps to enlighten whether m-learning suits the teaching-learning perspective. Besides this, plenty of presumptions have been tailored to clarify learners' adoption of m-learning and a definitive acceptance and implementation of it in a teaching-learning framework. Theories like “Diffusion of Innovation, the Theory of Reasoned Action, the Theory of Planned Behavior, the Technology Adoption Model (TAM), and the Unified Theory of Acceptance and Use of Technology (UTAUT)” have been restructured to reproduce m-learning's acceptance and implementation. These prototypes formulate the available outlook into aspects that put pressure on learners for the acceptance of m-learning. Learners' adoption has been characterized as “the eagerness in a learner's unit to utilize Information Technology for the jobs; it is intended to assist”. In the perspective of m-learning, learners' liking and recognition can be defined as the zeal in them to employ mobile technology to encourage their education.

The TAM model has been employed mostly in academic backgrounds to verify the implementation of instructional technology by instructors and learners. The TAM has to be customized and expanded to incorporate a variety of additional precursory variables to progress its extrapolative influence, including subjective norms, experience, and motivation. One of the supplementary variables that have been related with the TAM model is the scale of a learner's individual proficiency. Numerous researches have brought to light that a learner's individual proficiency for using computer technology is an important function in the acceptance of a broad variety of educational mediums. However, individual proficiency has only been looked at in a comparatively less research perspective on m-learning implementation.

## 2. Review of Literature

Mobile technologies have been regarded as potentially powerful enabling tool for educational progress. The widespread explanation of m-learning in academic literature is the employment of mobile technology with an Internet connection potential in education environment. The work in [8] stated this explanation plainly as “learning through mobile devices”. They added three essential prerequisites to this explanation. Foremost, the mobility factor connotes that learners are free to shift between and beyond multiple contexts and between subject matter and disciplinary contents and environments. Secondly, employing portable devices implies that education is not confined to formal learning environments; education is broadened inside informal prospects such as residence and work. Third, m-learning is not a one-way argument from teachers to learners, but constructive, understanding, and participating argument. The higher education institutions are on the lookout for technologies that suit the students needs effectively and encompass an efficient delivery medium [9]. The work in [10] states most people assess modernization, not on the foundation of scientific research by professionals, but by means of subjective evaluations of the quality of the modernization.

Acceptance of m-learning and its services by learners has been slow [11]. For instance, nonprofessional online students of the Open University in the United Kingdom had a tendency not to be supportive of m-learning due to expenses, battery life, technological complicatedness, and insufficient display sizes and interface [12]. On the contrary, the incorporation of m-learning services was intended to significantly develop easy access to educational courses and curriculum resources. To be precise, learners can, potentially, learn wherever and whenever, and the access of the mobile technology would better smooth the progress of instantaneous or real-time interactions between online learners and teachers [13].

Mobile use can put forward vast prospects for enhancing the education process among students. Yet, the benefits acquired from mobile usage rely on the intent of students to utilize them for learning purposes [14]. Technology Acceptance Model (TAM) widely used in the subject of Information Technology is employed to study the perceived usefulness and perceived ease of use (usability) as associated with learner's intent to utilize a technology or expertise [15]. Recently, TAM has been one of the widespread research theories that can facilitate an investigation for such intents and additionally categorize the influential and encouraging aspects for understanding the diverse emerging technologies [16].

According to [17], the aspects that impact the learners' acceptance to employ m-Learning through Short Message Service (SMS-learning) in the distance education in the Universiti Sains Malaysia (USM) are studied by adding usability as external factor. Survey was done by means of a questionnaire for 105 learners from management and sciences fields. Findings pointed out that the usability of the technology contributed to be effectiveness in assisting the learners with their learning. Learners had the same opinion that SMS-learning is simple, efficient, and useful for them in learning. On the other hand, the outcome exhibited that there has been a problem in m-learning that it reduced communication with teachers.

In Pakistan, a survey was performed by authors of [18] to uncover learners' insights about m-learning acceptance by employing facilitating circumstances, apparent playfulness, and societal influence as external variables on TAM model. Survey was performed with the respondents from 10 universities in Rawalpindi and Islamabad in Pakistan. The findings pointed out that apparent usefulness, usability, and facilitating circumstances drastically had an effect on the respondents' intent to accept m-learning, while apparent playfulness was found to have a smaller amount of persuasion. Societal influence was found to have a downbeat impact on acceptance of m-learning.

According to [19], in Taiwan, TAM model was broadened with perceived convenience as an external factor to scrutinize the consequence of apparent usefulness on the learners' outlook to employ mobile in English education. A survey was done with 158 college students from the middle part of Taiwan. The findings pointed out three essentials. First, perceived convenience, perceived usability, and apparent usefulness were antecedent variables that influence the adoption of English mobile education. Secondly, perceived convenience, perceived usability, and apparent usefulness had considerably a positive influence on the outlook toward employing mobile in English education. Third, the apparent usefulness and outlook toward employing mobile in English education had considerably a positive influence on the continuance of intent to utilize mobile.

In Jordan, authors of [20] carried out a research on an extended Technology Acceptance Model to explore the factors that influence the intent on m-learning. This research intention was to investigate the use of mobile phones in the learning situation and explore learners' anticipations and intents towards m-learning in Jordan. The model proposed was empirically tested using the data accumulated from a survey containing 21 variables. The researcher used a sample of 380 that were distributed randomly. The researcher established that every single factor significantly affected learners' behavioral intent except belief. Amongst all the factors, the perceived usability had the most significant effect.

In these studies, TAM has been extended with the external factors to explain and predict users' acceptance of M-learning in developing countries. The acceptance of m-learning is not similar in all countries due to the level of familiarity of the skill, accessibility of infrastructure, the proficiency in the new technology, and the motivation of the learners to put into practice and utilize the technology [21]. Furthermore, TAM set insight on studying why users' viewpoints and approaches affect their acceptance or rejection of Information Technology [22]. In spite of this extensive acceptance of mobile technology, there are only very few published studies to explore the acceptance of m-learning among Indian college students. This paper fills the gap in the literature by extending the classical TAM to illustrate how the perceived usability and the perceived usefulness of mobile technology would lay down the bond linking the learners' individual proficiency beliefs and the outlook of learners to use m-learning in future circumstances.

## 3. Theoretical Frame Work

Construction of the adoption method explains the aspects that influence a learner's views and usage of a particular expertise. One of the extensively used models to explicate adoption is the Technology Acceptance Model (TAM). The work in [15] devised the TAM to address the concern of how learners acknowledge and employ an expertise. The model identifies two variables, usability and usefulness, to be the basis of a learner's consent [23]. The TAM is a theoretical model used to model the underlying associations involving perceived usefulness and perceived usability and learner's objective and definite computer acceptance behavior [24].

Although TAM has been extensively used, it has been disapproved sometimes for not providing reliable and definite outcomes [25]. With the intent of tackling this denigration, [26] carried out a meta-analysis of experimental researches with the TAM. On the evaluation of 26 different studies, they came to a conclusion that the TAM presents itself as a fine way for deciding on the adoption of technology. Researchers established that there is an indication of an evident relationship between perceived usefulness and behavioral meaning and between perceived easy usability and perceived usefulness. However, the weaker association between the perceived easy usability and behavioral meaning suggested perceived usability functions in the course of the perceived usefulness. Substantiated on this discovery, [26] conceptualized a fresh model where perceived usability is moderated by perceived usefulness; but these alterations have not been extensively implemented. Regardless of these disapprovals, TAM has continued to be extensively applied and has revealed a fine analytical competence.

## 4. Individual Proficiency and M-Learning Acceptance

Individual proficiency points to the way individuals determine and decide with their own choices to make their effort continuously and the strongest eagerness that they experience when dealing with a particular work [27]. Individuals who have the high levels of individual proficiency have a greater chance of achieving a given work. Individual proficiency seems to be the sole factor that conforms what kind of activities they are involved in, the deep involvement in the activity, and the whole effort they show in any phases of adversities [28].

IT proficiency stems out from the Social Cognitive Theory of self-efficacy confidence [29]. IT proficiency, though it seems to be a subset, one's proficiency level, is described as the individual's final capability to use IT, as the proficiency is the measure of user's hopes to use, understand, and apply their will and skills.

The competency levels come out when the user has the skill of using multicomputing tools. Such users have goal achievement set up to resist the future negatives that may set in. These types of users have strong will to use this technology with the modern innovative tools to get the confidence of succeeding in their assignments. Users with a lower level of confidence lack the belief of using IT [30]. Previous experience and familiarity with the technology play a vital role. IT precisely refers to the individual's long term experience with the computers and the learning aptitude in terms of using newer applications. Individual proficiency has a positive relationship with the learner's previous experiences. This kind of belief has a strong base in Social Cognitive Theory.

Mobile learning adoption and individual proficiency show that the users' experience with mobile technology will improve their perceiving level of effort to achieve it, which is confirmed by many research studies. This plays a significant role in the field of individual proficiency and the learner's expectancy to continue to learn and achieve it. However, it is not a confirmed fact, as it may change from time to time and place to place [31].

Researches on the adoption of technology by the learning and the teaching population produce new factors for mobile learning. There is no such high level of study to give sufficient proof to the results arrived. This research has samples to find out the factors that make the acceptance of mobile learning by learners in Indian context to frame the relevant tools to model the acceptance of mobile learning by the learners.

## 5. Method of Investigation

The objective of this research is to illustrate how the perceived high usability and the perceived usefulness of mobile technology would stipulate the bond in linking the learners' individual proficiency beliefs and the outlook of learners to use m-learning in future circumstances. The populations of this research study were the final year university students. A multistage stratified convenience sampling technique was used for studying and selecting samples from three universities in three different geographical locations in India, by incorporating a mix of direct visits and computerized methods. A questionnaire was devised to evaluate the student's IT proficiency and outlook towards the incorporation of mobile based learning in their tertiary educational atmosphere.

Classes were randomly selected from the courses listed in the university academic calendars. Primarily, the respective Heads of the Departments were contacted seeking their consent to collect data from the students for the research. Of the students of 11 courses approached, students of two courses were not able to contribute as they were unavailable. Hence, two courses were chosen in addition to substituting them. Each group of students was briefed about the research study; students were then provided with the hard copy of a questionnaire and a website address to access the same questionnaire online. Students were given freedom to choose to complete the questionnaire either online or on the hard copy. Roughly, 41% of the students completed the provided questionnaire, and the rest did them online.

Students explored in this research study were from a variety of scholastic levels. Of the probable 2426 students requested to take part in the survey (based on the strength in each course given by the respective Head of the Departments), 892 students completed the questionnaire providing a response rate of 40%. Of the 892 respondents, 596 were Engineering students and the balance 296 were students from Arts and Science streams.

The resultant sample of the student set was satisfactory from a testing point of view. There was a diminutive concurrence on the quantity of responses suitable for Structural Equation Modeling [32]. However, [33, 34] advocating a sample of 200 in size would be appropriate for this form of statistical investigation. To mitigate the preconceived notion, it is suggested that research studies with “three or more indicators per factor, with a sample of 100, will regularly be enough for convergence and a sample of 150 will generally be adequate for a convergent and apt result” [35].

Though the sample was appropriately sized for the majority of statistics, it was not sufficient to allow a cross-validation of the structural equation model, as, dividing the sample size further, it would have produced a grouping excessively undersized to reliably compare [36]. Cross-validating is a quite difficult method of dividing a sample into two or more sets at random to run a comparison amidst the samples. This is used to verify the results arrived are stable among the samples [37].

## 6. Tool Deployment

The TAM was employed to find out the learners' expectations in acceptance of mobile education. The questionnaire comprised of 50 variables structured in a self-report manner associated with the research characteristics. The characteristics of TAM have been ascertained to verify the purpose to adopt [38, 39]. In this research study, perceived usefulness and perceived usability were evaluated by means of a 7-point Likert Scale rated from 1 to 7 with choice ordered from 1 (extremely likely) as the lowest to 7 (extremely unlikely) as the highest. The perceived usability characteristic is calculated if mobile usage was spotted to be liberated from the effort. The perceived usefulness characteristic calculated was that m-learning was believed as being advantageous to the education process. Questionnaire comprised of variables like the following: “will m-learning facilitate the frequent access to educational materials?” for perceived usefulness and “to get comfortable with M-learning is a time consuming process” for perceived usability. One query was employed to obtain the respondents' future objective to adopt m-learning. The respondents replied with the following: “on the whole, I believe m-learning is likely to be valuable to my education and I am probably ready to accept it, if I have any prospects in the future”.

**Figure 1:**
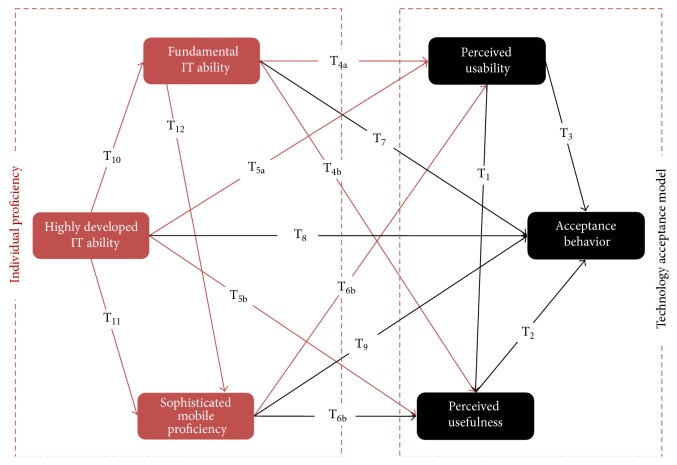
Model of students' acceptance of m-Learning.

The model in Figure 1 explains the causal relationships between perceived usefulness, perceived usability, acceptance behavior, fundamental IT ability, highly developed IT ability, and sophisticated mobile proficiency. The proposed model based on TAM model will be described depending on three particular principles, perceived usefulness, perceived usability, and acceptance behavior that will be determined by external variables: fundamental IT ability, highly developed IT ability, and sophisticated mobile proficiency.

The learners' expectations in acceptance towards mobile education will be determined by three particular aspects: perceived usefulness, perceived usability, and acceptance behavior. Based on the above theoretical variables, this study presents research model and will discuss the bond linking the learners' individual proficiency beliefs and the outlook of learners to use m-learning in future circumstances. This study also establishes linkage between these sets of variables as found in the proposed model illustrated in Figure 1 which becomes the basis for framing the following hypothesis.

*Based on the TAM model, three hypotheses were analyzed*:

 T_1_: learners who identify m-learning usability as uncomplicated tend to have an optimistic opinion of the utility of m-learning.
 T_2_: learners who recognize m-learning as constructive are further expected to point out that they are determined to accept mobile expertise in the learning process.
 T_3_: learners who point out that m-learning usability is high are more liable to specify that they plan to take up mobile expertise in the learning process.

The IT proficiency scale was created enlisting various technological skills. Respondents were requested to grade their ability on all skills. The research study carried out was based on [40] that consisted of determining the unanimously employed technology-based activities of a learner. The actual research contained a survey with 38 variables that were divided into eight groups.

A trial study was done to condense the variables to 16 crucial skills that were related to both the IT and mobile proficiency in the learners. IT based skills required a series of abilities from using word processing software to explore and download contents from the World Wide Web. Mobile technology-based skill consisted of variables concerning skill sets such as sending and receiving texts to download programs to their mobile devices. The proficiencies were evaluated on a 7-point scale: 1 = extremely hard, 7 = extremely easy. This research will thus investigate the connection that IT expertise has on m-learning implementation.

Specifically, the following hypotheses will be analyzed:

 
$T_{4\text{–}6}$: learners with advanced state of the essential IT proficiency (T_4_), higher IT skills (T_5_), and/or highly developed mobile proficiency (T_6_) are highly liable to find the usability of m-learning as uncomplicated and constructive.
 
$T_{7\text{–}9}$: learners with advanced state of the essential IT proficiency (T_7_), higher IT skills (T_8_), and/or highly developed mobile proficiency (T_9_) are very much expected to take on m-learning.

In addition, the following relationships were tested:

 
$T_{10\text{–}12}$: if the learners are trained in one aspect of the IT, they will be highly prone to take up a broader variety of IT skills.

## 7. Data Analysis

During the study, the researchers applied “multivariate” conceptual application techniques to analyze the data. A valid construct degree was adopted that represented the theoretical concept. Convergent, divergent, and discriminant validities exist that are referred to as the subcategories of construct for its validity [41]. The research study extracted the latent constructs from the 12-variable measure for the IT skills by employing Explanatory Factor Analysis (EFA). These 12 variables were grouped into four groups, based on the EFA, which seem to be the ability to use fundamental IT skills, highly developed IT skill, and sophisticated mobile proficiency every day. Three variables were taken out to represent each construct in each category. During the analysis, the variables taken out had high > 0.8 as consistent. Also, the skills associated with general computing tasks such as using office automation software, browsing on the World Wide Web, and basic mobile activities such as messaging, voice-calling, and sharing pictures were included.

The efficiency of users in the field of advanced computing such as using a chat room was addressed using the highly developed IT abilities. Three strong variables were taken out to represent each construct. The EFA results showed that two constructs were for the perceived usefulness and the other two were for the easy usage of mobiles.

The total accuracy approach was adopted to calculate the estimates using Cronbach's alpha method. The range of accuracy of the constructs remained between 0.73 and 0.95 exceeding the threshold of 0.8 [42]. Structural Equation Modeling (SEM) was used to interpret and analyze the results. This method enabled the analysis of the nature of casual relationships among the variables.

Totally, 892 students participated in the research study. Of the total 892 respondents, there were 669 (75%) males and 223(25%) females. The average student's age was between 19 and 25 years. All the participants were Indian citizens.

## 8. Results

**Figure 2:**
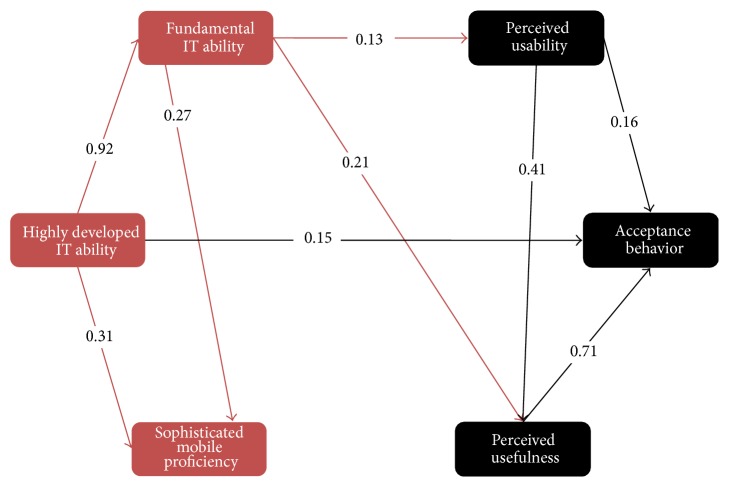
Results for SEM modeling on students' acceptance of m-Learning.

To examine the impact that an expert has on perceived usability, perceived usefulness, and behavioral intention, the Structural Equation Modeling was used. All the important consistent path coefficients are shown in Figure 2. This model was identified as a good fit by analyzing the goodness of fit statistics (*χ* ^2^ = 4.82, df = 7, *P* < 0.605, SRMR = 0.03, TLI = 0.95, CFI = 0.96, PCFI = 0.43, and RMSEA = 0.0 (91% CI = 0.00–0.05). Only *χ* ^2^ *P* value of all the fit statistics expressed a good fit. Despite the fact that Chi-square (*χ* ^2^), its degrees of freedom (df), and *P* value are presented, a baseline indication of model fit, *χ* ^2^, has been publicized to be a weak marker of model fit, repeatedly generating incorrect negatives [37].

The student adoption model confirmed that assessment of usability and the utility of m-learning had a dominant effect on learners' objective to accept and implement m-learning. Fine fundamental IT abilities are powerfully related with sophisticated mobile proficiency and also with highly developed IT skills. The model, moreover, ascertains the less significant role of the degree of fundamental IT proficiency in impacting their perception to accept and implement m-learning. The findings signify that learners who are highly proficient in a range of fundamental IT activities were highly prone to accept and implement m-learning. Sophisticated mobile proficiency was publicized to arbitrate the task of perceived usability and perceived usefulness on the objective of learners to accept and implement m-learning. This specifies that learners' skill level of mobile expertise will impact their opinion on m-learning.

## 9. Discussion

Earlier investigation has constantly accounted for a constructive association linking precedent IT understanding and individual proficiency viewpoints [43]. On the other hand, these researches have not deemed the influence that diverse kinds of prior proficiency may have on the opinion of usability and constructiveness for a specific skill. In this research study, three categories of IT skills were acknowledged, fundamental IT skills, highly developed (proficient) IT skills, and sophisticated mobile proficiency, and assessed for their acceptance and implementation level of m-learning.

The research study illustrates that perceived usefulness of mobile technology had the most resourceful effect on the objective to accept m-learning, tagged with a minor degree of the perceived usability. However, usability had a supplementary implied influence on the objective of acception through its solid impact on perceived usefulness. This implies that if learners are to accept and implement m-learning, they should perceive it as easy to employ and trust that it puts forward major advantages above the offered educational routines.

The research study also illustrates that the impact of expertise and knowledge with mobile skill on the acceptance and implementation of m-learning is intervened through the influence of perceived usefulness and the perceived usability. This implies that learners with a considerable point of knowledge in the supplementary sophisticated characteristics of mobile expertise will be mutually relaxed with employing it for education in addition to the advantages it puts forward in encouraging their education. The effect of mobile expertise knowledge on m-learning acceptance and implementation has just been concentrated in a small number of researches [44]. This result validates that the perceived notions developed based on the earlier experience of using a particular technology impact the potential acceptance and implementation of that skill [45].

The effect that knowledge has on perceived usefulness advocates that learners are proficient to assess more precisely how important m-learning will be in augmenting their education. Acquaintance with a particular expertise will facilitate a backing for the expansion and testing with an associated type of expertise. Concurrently, learners grow to be more accustomed with the skill they find that can be used in many ways. Particularly, as the learners become more trained in employing mobile technology, they will be highly induced to search for fresh mobile services. A learner who rarely makes use of mobile devices or has a depleted amount of knowledge with using the technology will more likely not try out or differ from the present usage and be less prone to accept m-learning as simple to handle. In contrast with inexperienced and hesitant learners, assertive learners are highly prone to maximize the utilization of the mechanism and discover latest techniques and methods to enhance the effectiveness of the mechanism. This backs [46] who established that educationalists who turn out to be more accustomed with their mobile mechanisms acquired a better insight of developing and integrating m-learning, in the teaching-learning process.

It was established that learners who were proficient fundamental IT users were highly inclined to accept m-learning. The research study illustrated that the fundamental IT proficiency and the objective to accept and implement m-learning, not interceded by either the perceived usability or the perceived usefulness, were directly related. This result elucidates, for the first instance, the effect of fundamental IT proficiency on m-learning acceptance and implementation, an association that has not formerly been investigated in related works. This proposes that the objective to accept and implement a new skill is improved by analyzing the low skill levels of individuals in a variety of essential IT skills. These findings emphasize the significance of the function that fundamental IT proficiency plays in the acceptance and implementation of new set of skills. It indicates that learners with fundamental IT proficiency are highly likely to recognize acquiring new skills as constructive and implement them in the future. This conception is backed up by the strong connection linking the fundamental IT skills, the sophisticated mobile proficiency, and the highly developed IT skills. Fundamental IT skills appear to be a significant aspect for building a fresh or sophisticated proficiency in other skills. The presentation of an explicit expertise may be of additional use if the proposed learners had a fundamental foundation in common IT skills.

Although the explicit associations amid at the present skill and the objective to impart a new skill were established, the model too proposes that being proficient in one domain endorses the maturity of acquiring knowledge in other domains.

Well accomplished learners in one domain were expected to be very much talented in other domains. The encasing of abilities illustrate that trust and positive understanding of IT will develop confidence in individuals, such that the learners obviously intend to gain knowledge of a fresh expertise as they obtain additional knowledge with their inherent talents. On the other hand, outside the effect of a fundamental degree of IT and a sophisticated level of mobile proficiency, no added pressure is laid on the learner to take up a fresh expertise evident.

After organizing the learners for m-learning, a broader convergence is required on how IT is integrated and presented. Educationalists must be conscious to facilitate imparting the learners with a fundamental amount of IT expertise before the learners are led into m-learning. In view of the fact that a lot of actions preformed on a mobile device are as well carried out on an IT device, it might be valuable to study how to efficiently accomplish them easier on an IT device, prior to making headway to a mobile setting. For instance, exchanging mails and surfing the World Wide Web are mutually feasible employing an IT device and a mobile device. If a learner realizes that executing these actions on an IT device is easy and relaxing, it may possibly be not as much complex to accomplish these actions on a mobile setting.

This research study uncovered no proof that highly developed IT skills impacted the objective to accept and implement m-learning. The discovery that highly developed IT skills did not play an important part in the opinion of perceived usability or perceived usefulness as fascinating and fresh. It additionally toughens the case that fundamental IT skills and the skills in any allied expertise are vital bases for the acceptance and implementation of m-learning. This finding implies that highly developed expertise in unconnected skills encompasses no perceptible effect on the acceptance and implementation of an explicit skill. Earlier studies do not appear to have probed the association among the particular sections of capability and acceptance. In spite of this, additional investigation is essential to establish whether these relationships shall be instituted in further circumstances to elucidate the method that clarifies them.

## 10. Conclusion

The endeavor of acquiring a modern skill is a costly and lengthy scheme, and the chances of failure are likely, if not rightly, judged. The frequent attempts to establish modern skills fall short to judge learner outlooks, the risk of wasting valuable work, and reserves and have a possibility of failure to understand the full advantages of the modern skill [47]. Hence, learners' approval is a key feature in influencing the implementation of m-learning, and in tertiary learning, the achievement of m-learning depends upon learners' appreciation of the advantages of acquiring the skill. Studies have revealed that the opinions of learners will play a significant role in the achievement of whichever innovative project implemented, as it is the fact that the learners are the ones who will be making use of the expertise in their education [48]. This research has underlined the effect of definite prior proficiency and knowledge on learners' opinions on mobile skill. It illustrates that learners with strong fundamental IT proficiency display a reasonably elevated intention to accept m-learning. Learners with superior mobile device proficiency are highly likely to pick out m-learning as simple-to-employ and helpful method of learning. This research hence points to the significance of foreseeing learners' pessimistic approaches branching from low IT skills (individual proficiency). By imparting and encouraging IT skills, learners can be facilitated to make m-learning appear less discouraging and persuade its implementation. Consistent support and assistance are perceived as concurrently important to maintain the simplicity of learning and retain the eagerness of learners to experiment and acquiring mobile skills.

This research study proposed to ascertain a few of the basics of m-learning, for more perceptive influences on the acceptance of the same. Acceptance was established through earlier pilot researches made on m-learning and fresh conclusions were arrived too. Nevertheless, this was a transversal study and observational research vital to institute the results of associations more definitely. Besides, the research was supported by the TAM. The TAM originated from the situation where learners had previously employed the selected skill and subsequently envisaged its potential usage. This research did not presume that learners had any knowledge of m-learning, however, banked on the learners' understanding of the content with their inherent mobile skills. Learners were likely to direct the perceptiveness of their mobile skill to the circumstances of employing it for education. This method of building up a mobile education implementation version supported on restricted understanding is not novel and a number of researches have employed this in identical circumstances [49]. Besides, future usage was analyzed from a confirmed objective to accept and implement m-learning. Extensive experimental studies have defined the fundamental connection linking the objective to acceptance, and the concrete prospect of acceptance and implementation, consequently offering some confidence to the use of physiological intention as a pointer of the definite potential acceptance and implementation [50].

To sum up, although this research study is not devoid of the limitations, the techniques implemented have effects in arriving at considerable conclusions on a part that is fresh and rising with insufficient experiential studies. Overall, yet in view of the constraints, these conclusions affix considerable worth to our perception of m-learning espousal.

## Figures and Tables

**Figure 3:**
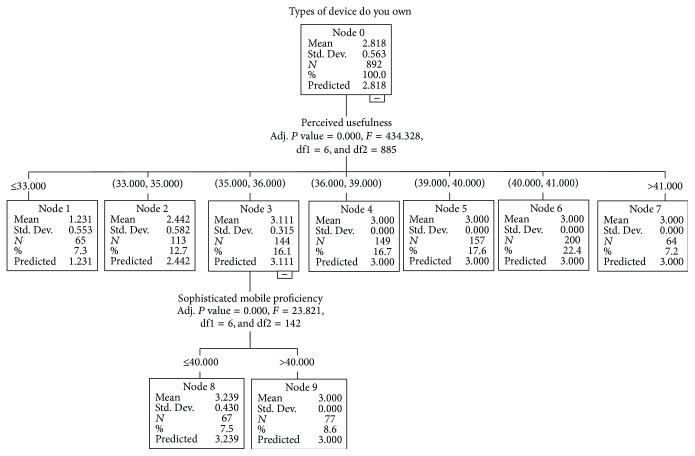


**Table 1:** 

Notes	
*Comments*	
Input	
Data	C:∖Users∖data.sav
Active dataset	DataSet1
Filter	<none>
Weight	<none>
Split file	<none>
*N* of rows in working data file	892
Missing value handling	
Definition of missing	Handling of user-defined missing values of nominal independent variables depends on the growing method.
Cases used	Only cases with valid data for the dependent variable and some or all independent variables are used in computing any statistics.
*Syntax*	TREE device [s] BY USEFULNESS [s] USABILITY [s] ACCEPTENCE [s] BASIKSKILL [s] ADVANCESKILL [s] MOBILESKILL [s]
	/TREE DISPLAY=TOPDOWN NODES=STATISTICS BRANCHSTATISTICS=YES NODEDEFS=YES SCALE=AUTO
	/PRINT MODELSUMMARY RISK
	/GAIN SUMMARYTABLE=YES TYPE=[NODE] SORT=DESCENDING CUMULATIVE=NO
	/METHOD TYPE=CHAID
	/GROWTHLIMIT MAXDEPTH=AUTO MINPARENTSIZE=100 MINCHILDSIZE=50
	/VALIDATION TYPE=NONE OUTPUT=BOTHSAMPLES
	/CHAID ALPHASPLIT=0.05 ALPHAMERGE=0.05 SPLITMERGED=NO ADJUST=BONFERRONI INTERVALS=10.
Resources	
Processor time	00:00:00.90
Elapsed time	00:00:00.59
Files saved	
Rules file	

Model summary	

Specifications	
Growing method	CHAID
Dependent variable	Types of device you own
Independent variables	PERCEIVED USEFULNESS, PERCEIVED USABILITY, ACCEPTANCE BEHAVIOR, FUNDAMENTAL IT ABILITY, HIGHLY DEVELOPED IT ABILITY, SOPHISTICATED MOBILE PROFICIENCY
Validation	None
Maximum tree depth	3
Minimum cases in parent node	100
Minimum cases in child node	50
Results	
Independent variables included	PERCEIVED USEFULNESS, SOPHISTICATED MOBILE PROFICIENCY
Number of nodes	10
Number of terminal nodes	8
Depth	2

**Table 2:** Gain summary for nodes.

Node	*N*	Percent	Mean
8	67	7.5%	3.24
6	200	22.4%	3.00
5	157	17.6%	3.00
4	149	16.7%	3.00
9	77	8.6%	3.00
7	64	7.2%	3.00
2	113	12.7%	2.44
1	65	7.3%	1.23

Growing method: CHAID.

Dependent variable: types of device you own.

**Table 3:** Risk.

Estimate	Std. error
0.078	0.010

Growing method: CHAID.

Dependent variable: types of device you own.

**Table 4:** 

	Estimate	SE	CR	*P*
Perceived usefulness ← acceptance behavior	0.103	0.045	7.750	0.16
Perceived usefulness ← perceived usability	0.331	0.036	11.275	0.41
Acceptance behavior ← perceived usability	0.018	0.047	1.279	0.71
Fundamental IT ability ← perceived usefulness	0.181	0.045	−4.230	0.21
Fundamental IT ability and sophisticated mobile proficiency	−0.075	0.057	5.092	0.27
Fundamental IT ability ← highly developed IT ability	−0.041	0.052	−2.460	0.92
Highly developed IT ability ← sophisticated mobile proficiency	0.043	0.053	10.931	0.31
Highly developed IT ability ← acceptance behavior	0.119	0.046	6.200	0.15

**Table 5:** Model summary and parameter estimates. Dependent variable, perceived usefulness.

Equation	Model summary					Parameter estimates		
	*R* square	*F*	df1	df2	Sig.	Constant	*b*1	*b*2
Linear	0.010	10.379	1	998	0.001	3.432	0.094	—
Quadratic	0.012	5.936	2	997	0.003	3.709	−0.086	0.027

The independent variable is acceptance behavior.

## Appendices

### A. Classification Tree

See Tables 1, 2, and 3 and Figure 3.

### B. Regression Weights

See Tables 4 and 5.
